# Atorvastatin modulates the expression of aging-related genes in the brain of aging induced by D-galactose in mice

**DOI:** 10.22038/IJBMS.2021.58502.12996

**Published:** 2021-10

**Authors:** Manijeh Motevalian, Neda Tekyeh Maroof, Mohammad Hadi Nematollahi, Fatemeh Khajehasani, Iman Fatemi

**Affiliations:** 1 Razi Drug Research Center, Iran University of Medical Sciences, Tehran, Iran; 2 Department of Pharmacology, School of Medicine, Iran University of Medical Sciences, Tehran, Iran; 3 Department of Clinical Biochemistry, Kerman University of Medical Sciences, Kerman, Iran; 4 Department of Radiology, Afzalipour Faculty of Medicine, Kerman University of Medical Sciences, Kerman, Iran; 5 Research Center of Tropical and Infectious Diseases, Kerman University of Medical Sciences, Kerman, Iran

**Keywords:** Aging, Animal model, Apoptosis, Gene expression, Inflammation

## Abstract

**Objective(s):**

Atorvastatin (AT), a competitive inhibitor of 3-hydroxymethyl-3-glutaryl-coenzyme-A reductase, is a cholesterol-lowering drug. AT has been shown to have neuroprotective, antioxidant, and anti-inflammatory properties. Previously, we have reported that AT could attenuate the behavioral, renal, and hepatic manifestations of aging. To clarify further the mechanisms involved, the present study was designed to evaluate the effect of AT on the expression of some aging-related genes in the brain of aging mice induced by D-galactose (DG).

**Materials and Methods:**

For this purpose, AT (0.1 and 1 mg/kg/p.o.) was administrated daily in DG-received (500 mg/kg/p.o.) mice model of aging for six weeks. At the end of the experiment, mice were decapitated to remove the brains. Then, the expression profiles of sirtuin 1 (Sirt1), P53, P21, Bcl-2, Bax, superoxide dismutase (SOD), catalase (CAT), glutathione peroxidase (GPx), interleukin 1 beta (IL1β), tumor necrosis factor-alpha (TNFα), inducible nitric oxide synthase (iNOS), cyclooxygenase-2 (COX-2), and brain-derived neurotrophic factor (BDNF) were assessed using the real-time PCR method.

**Results:**

The present study shows that DG decreases the expression of Sirt1, Bcl-2, CAT, GPx, and BDNF while increasing the expression of P53, P21, Bax, IL-1β, iNOS, COX-2, and TNF-α. According to the findings of the present study, AT (more potentially at the dose of 1 mg/kg) modulates the expression of these aging-related genes in the brain of aging mice.

**Conclusion:**

The results of the present study confirmed our previous reports on the anti-aging effects of AT at the gene level, the precise mechanisms and underlying pathways need further studies.

## Introduction

According to the United Nations reports, the population of elderly (over 65 years) people rises to 21.5% of the global population by 2050 (1). Aging is a time-dependent phenomenon leading to progressive physiological dysfunction and reduced quality of impairments. Behavioral impairment such as anxiety, depression, and different aspects of cognitive impairments are the most important features of brain aging (3, 4). The exact underlying mechanisms of aging and age-related neurologic diseases are not well understood, but the suggested theories are: decline in neurotransmitters’ levels, changes in brain structural connectivity, oxidative stress, and lipid peroxidation (5, 6). Thus, investigation on the pathophysiology of aging and finding a method to postpone these processes could be beneficial for medical gerontology (7, 8).

D-galactose (DG)-induced accelerated aging model has demonstrable reliability, predictive validity, construct validity, and relevance (9, 10). Long-term administration of DG recapitulates many features of aging in various tissues and has been extensively applied to investigate the mechanisms of aging in rodents (7, 11, 12).

Statins such as atorvastatin (AT) act via inhibiting the 3-hydroxy-methyl-glutaryl coenzyme A reductase enzyme which is an important enzyme in the biosynthesis of cholesterol (13). AT is widely prescribed for atherosclerotic and hypercholesterolemia patients (14). On the other hand, other pharmacological properties have been reported for AT such as neuroprotective, anti-inflammatory, and antioxidant activities (15). We confirmed that AT could reduce the aging behavioral manifestations in aging induced by DG in mice such as anxiety, sarcopenia, and cognitive impairments (16). In another recent study, we also demonstrated that AT could attenuate the dysfunction of the liver and kidney in aging mice (17).

In order to further clarify the aging phenomenon and translate the findings from bench to bedside, the present study was designed to evaluate the effect of AT on the expressions of some aging-related genes in the brain of aging mice induced by DG.

## Materials and Methods

***Animals***

The experiments were performed on 24 male mice weighing 18–22 g. Animals were kept at 25±1 ^°^C on a 12-hr light/dark cycle with free access to food (pellet chow) and water. Three mice were housed in standard polypropylene cages with wired-net top in a controlled room. All efforts were made to minimize the number of animals used and their suffering. It should be considered that animal housing, surgery, and testing rooms (22±2 ^°^C) were close together and the animals were transported between the rooms in their home cages. Behavioral tests and animal care were conducted in accordance with the standard ethical guidelines (NIH, publication No. 85-23, revised 1985; European Communities Directive 86/609/EEC) and approved by the Local Ethical Committee (IR.IUMS.REC.1395.95-04-118-30197).

***Drugs***

AT (Lipitor™) was purchased from Pfizer Pharmaceuticals (USA). DG was purchased from Sigma Aldrich (Germany). 

***Experimental procedures and treatment***

After 2 weeks of acclimatization, mice were randomly divided into four groups as follows: control, aging, AT 0.1, and AT 1 (Table 1). The dosages and route of administration for AT and DG were selected from previous works (18-21).

***Tissue preparations***

Twenty four hr after the last administration of AT, mice of all groups were sacrificed and their brains were quickly removed. The brains were immediately removed under aseptic condition and both hemispheres were stored at -80 ^°^C until further analysis.

***RNA extraction and real-time RT-PCR***

We conducted real-time RT-PCR to examine the effects of DG on mRNA expressions of sirtuin1 (Sirt1), P53, P21, Bcl-2, Bax, superoxide dismutase (SOD), catalase (CAT), glutathione peroxidase (GPx), interleukin 1 beta (IL-1β), tumor necrosis factor-alpha (TNF-α), inducible nitric oxide synthase (iNOS), cyclooxygenase-2 (COX-2), and brain-derived neurotrophic factor (BDNF) in the brain of different experimental groups. Total RNA was extracted from frozen mice brain using TRIZOL Reagent (U.S. Patent No. 5, 346, 994) according to the manufacturer’s instructions. After normalization of all extracted RNA to 1000 ng/ml, RNA was reverse transcribed into single-strand cDNA using Takara kit (Takara, Japan) following the protocols. The quantity and purity of extracted RNA were analyzed using Nano-Drop (Technologies, ND-2000). The product used for quantitative RT-PCR using cyber green/ROX (Takara, Japan) is based on the protocol of bioneer exicycler 96 qRT-PCR thermal cycler (Korea, bioneer). The amplification protocol comprised of 1 cycle at 95 ^°^C for 2 min followed by 40 cycles at 95 ^°^C for 15 sec, 60 ^°^C for 30 sec, and then 72 ^°^C for 10 sec. The relative expression of the studied genes to the housekeeping gene was calculated by measuring the 2^-ΔΔCt^ value for each sample. Table 2 illustrated the primers used in RT-PCR. Nicotinamide adenine dinucleotide phosphate (NAPDH), as a housekeeping gene, was used to normalize the amplified signals of the target genes.

***Statistical analysis***

Statistical analysis was performed using GraphPad Prism software (version 6). All results are expressed as mean±SEM. The normality of the results was tested using the Kolmogorov-Smirnov test. For parametric data, the comparisons were made by one-way ANOVA followed by Tukey’s *post hoc* test and for non-parametric data, the comparisons were made by Kruskal-Wallis followed by Dunn’s *post hoc* test. *P*-values<0.05 were considered statistically significant.

## Results

***The effect of AT on the aging-related genes expression***

**Figure 1:**
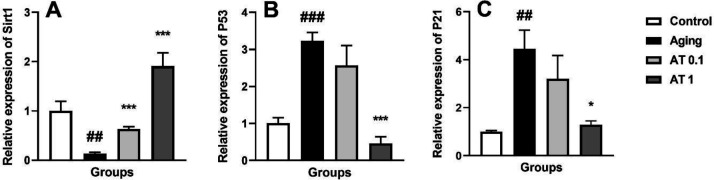
Effect of atorvastatin on the expressions of Sirt1 (A), P53 (B) and P21 (C) in aging mice (n=6). ^##^*P<*0.01 and ^###^*P<*0.001 as compared with the control group; ^*^P<0.05 and ^***^P<0.001 as compared with the aging group

The results of aging-related genes expression are illustrated in Figure 1. DG-induced aging significantly reduced the expression of Sirt1 mRNA compared with the control group (F (3, 20)=19.61; *P*<0.01) (Figure 1A). Administration of AT (0.1 and 1 mg/kg) in aging mice increased the expression of Sirt1 mRNA compared with the aging group (all *P*<0.001).

Moreover, aging increased the expression of P53 mRNA compared with the control group (F (3, 20)=17.67; *P*<0.001) (Figure 1B). Oral administration of AT at the dose of 1 mg/kg in aging mice significantly decreased the expression of P53 mRNA in comparison with the aging group (*P*<0.001).

Furthermore, aging induced by GD increased the expression of P21 mRNA in comparison with the control group (*P*<0.01) (Figure 1C). Administration of AT (1 mg/kg) in aging mice decreased the expression of P21 mRNA compared with the aging group (*P*<0.05). 

***The effect of AT on the apoptosis-related gene expression***

**Figure 2:**
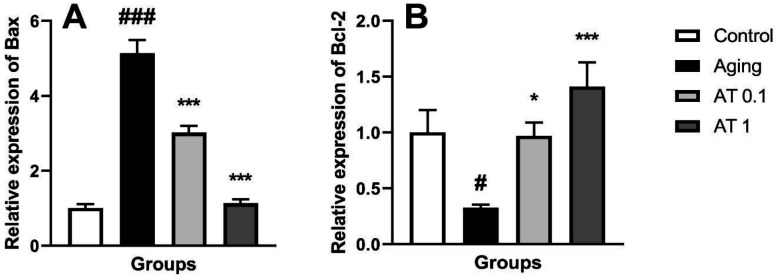
Effect of atorvastatin on the expressions of Bax (A) and Bcl-2 (B) in aging mice (n=6). ^#^*P<*0.05 and ^###^*P<*0.001 as compared with the control group; ^*^P<0.05 and ^***^P<0.001 as compared with the aging group

The results of apoptotic gene expression were illustrated in Figure 2. Aging significantly increased the expression of Bax compared with the control group (F (3, 20)=1.44; *P*<0.01) (Figure 1A). AT at both doses decreased the expression of Bax compared with the aging group (all *P*<0.001). 

Moreover, aging decreased the expression of Bcl-2 mRNA compared with the control group (F (3, 20)=6.09; *P*<0.05) (Figure 1B). Oral administration of AT at doses of 0.1 and 1 mg/kg in aging mice significantly increased the expression of Bcl-2 mRNA in comparison with the aging group (*P*<0.05 and *P*<0.001, respectively).

***The effect of AT on the antioxidant-related genes expression***

**Figure 3:**
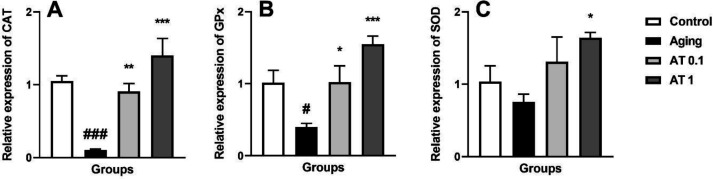
Effect of atorvastatin on the expressions of CAT (A), GPx (B), and SOD (C) in aging mice (n=6). ^#^*P<*0.05 and ^###^*P<*0.001 as compared with the control group; ^*^P<0.05, ^**^P<0.01 and ^***^P<0.001 as compared with the aging group

The results of antioxidative gene expression are illustrated in Figure 3. Aging significantly decreased the expression of CAT (F (3, 20) = 16.98; *P*<0.001) and GPx (F (3, 20)=9.239; *P*<0.001) mRNA in comparison with the control group but has no significant effect on SOD mRNA expression (F (3, 20)=3.180; *P*<0.001). Administration of AT (0.1 mg/kg) in DG-treated animals increased the expression of CAT (*P*<0.01) and GPx (*P*<0.05) mRNA compared with the aging group. Moreover, AT at the dose of 1 mg/kg significantly increased the expression of CAT (*P*<0.001), GPx (*P*<0.001). and SOD (*P*<0.05) mRNA in comparison with aging animals.

***The effect of AT on inflammatory-related gene expression***

**Figure 4:**
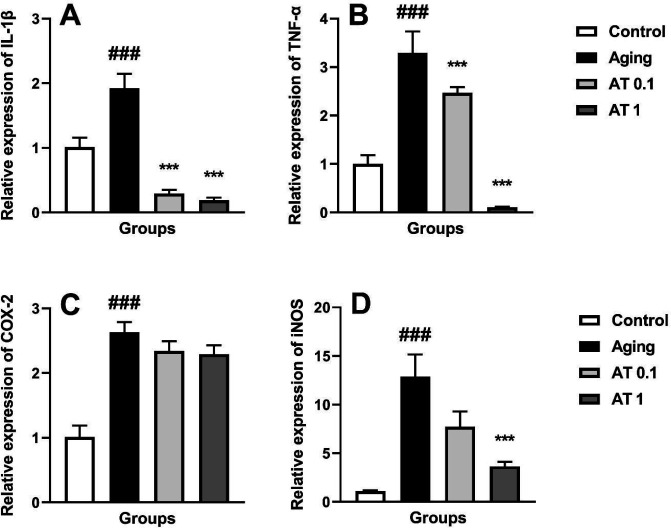
Effect of atorvastatin on the expressions of IL-1β (A), TNF-α (B), COX-2 (C) and iNOS (D) in aging mice (n=6). ^###^*P<*0.001 as compared with the control group; ^***^P<0.001 as compared with the aging group

The results of inflammatory gene expression are illustrated in Figure 4. Aging induced by DG increased the expression of IL-1β (F (3, 20)=33.45; *P*<0.001), TNF-α (F (3, 20)=34.15; *P*<0.001), COX-2 (F (3, 20)=20.81; *P*<0.001), and iNOS (F (3, 20)=13.57; *P*<0.001) mRNA compared with the normal animals. Moreover, oral administration of AT at the dose of 0.1 mg/kg in DG-treated animals increased the expression of IL-1β and TNF-α mRNA compared with aging animals (all *P*<0.001). Furthermore, AT (1 mg/kg) significantly increased the expression of iNOS, IL-1β and TNF-α mRNA in comparison with the aging group (all *P*<0.001).

***The effect of AT on the BDNF gene expression***

**Figure 5:**
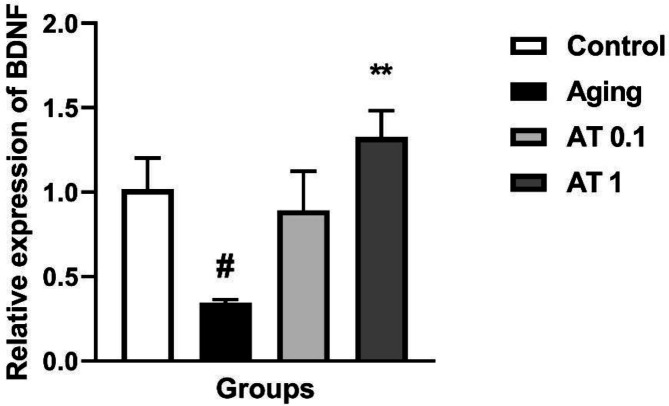
Effect of atorvastatin on the expressions of BDNF in aging mice (n=6). ^###^ significant difference in comparison with control group (n=6). ^#^*P<*0.05 as compared with the control group; ^**^P<0.01 as compared with the aging group. AT: atorvastatin. 0.1 and 1 imply 0.1 and 1 mg/kg

Administration of DG significantly decreased the expression of BDNF mRNA compared with the normal animals (F (3, 20)=5.968; *P*<0.05) (Figure 5). Administration of AT with the higher dose (1 mg/kg) increased the expression of BDNF mRNA in aging mice compared with aging animals (*P*<0.01).

**Table 1:** The different experimental groups (n=6)

Group	Intervention
Control	Healthy normal animals without any intervention
Aging	Received DG at the dose of 500 mg/kg per 10 ml drinking water for 6 weeks
AT 0.1	Received DG at the dose of 500 mg/kg per 10 ml drinking water plus AT 0.1 mg/kg/day intragastrically for 6 weeks
AT 1	Received DG at the dose of 500 mg/kg per 10 ml drinking water plus AT 1 mg/kg/day intragastrically for 6 weeks

**Table 2:** The primers used for the gene expression in the brains of aging mice

Sirt1	Forward: 5′-ATGTGAGGAGTCAGCACCGT-3′ (sense) Reverse: 5′-TAGTCTCAGGGGCCTGTTTG-3′(antisense)
Bax	Forward: 5′-TGCTACAGGGTTTCATCCAGG-3′ (sense) Reverse: 5′-CATATTGCTGTCCAGTTCATCTC-3′(antisense)
Bcl-2	Forward: 5′-ACTTCTCTCGTCGCTACCGT-3′ (sense) Reverse: 5′-ATAGTTCCACAAAGGCATCCCA-3′(antisense)
P21	Forward: 5′-GACAAGAGGCCCAGTACTTC-3′ (sense) Reverse: 5′-GCTTGGAGTGATAGAAATCTGTC-3′(antisense)
P53	Forward: 5′-TGCTCACCCTGGCTAAAGTT-3′ (sense) Reverse: 5′-AATGTCTCCTGGCTCAGAGG-3′(antisense)
COX-2	Forward: 5′-GCCTACTACAAGTGTTTCTTTTTG -3′ (sense) Reverse: 5′-CATTTTGTTTGATTGTTCACACCAT-3′(antisense)
iNOS	Forward: 5′-CTTGCCCCTGGAAGTTTCTC-3′ (sense) Reverse: 5′-CAAGTGAAATCCGATGTGGC-3′(antisense)
IL-1β	Forward: 5′-TGCCACCTTTTGACAGTGATG-3′ (sense) Reverse: 5′-GGTCCACGGGAAAGACAC-3′(antisense)
TNF-α	Forward: 5′-GAACTGGCAGAAGAGGCACT-3′ (sense) Reverse: 5′-TTGAGAAGATGATCTGAGTGTGAG-3′(antisense)
SOD	Forward: 5′-TCGTCTTGCTCTCTCTGGTCC-3′ (sense) Reverse: 5′-GGTTCACCGCTTGCCTTCTG-3′(antisense)
CAT	Forward: 5′-CAATGTCACTCAGGTGCGGA-3′ (sense) Reverse: 5′-CTTAGGCTTCTCAGCGTTGTAC-3′(antisense)
GPx	Forward: 5′-CCACCGTGTATGCCTTCTCC-3′ (sense) Reverse: 5′-GATCGTGGTGCCTCAGAGAG-3′(antisense)
BDNF	Forward: 5′-GTGACCTGAGCAGTGGGCAAAG-3′ (sense) Reverse: 5′-ATATAGCGGGCGTTTCCTGAAGC-3′(antisense)
NAPDH	Forward: 5′-GAAGATTTGCCTGGAAGAACC-3′ (sense) Reverse: 5′-AGGTTTGTTGCTCCTGATGC-3′(antisense)

AT: atorvastatin. 0.1 and 1 imply 0.1 and 1 mg/kg

AT: atorvastatin. 0.1 and 1 imply 0.1 and 1 mg/kg

AT: atorvastatin. 0.1 and 1 imply 0.1 and 1 mg/kg

AT: atorvastatin. 0.1 and 1 imply 0.1 and 1 mg/kg

## Discussion

The results of the present study imply that administration of D-gal at the dose of 500 mg/kg per 10 ml drinking water for 6 weeks, causes severe aging-related changes, including a significant decrease in mRNA expression of Sirt1, Bcl-2, CAT, GPx, and BDNF as well as an increase in mRNA expression of P53, P21, Bax, IL-1β, iNOS, COX-2, and TNF-α. However, AT (more potentially at the dose of 1 mg/kg) could attenuate these deteriorating effects of aging.

The expression levels of Sirt1, P53, and P21 are closely associated with mammalian aging and emerged as important indicators in the natural aging and aging model induced by D-gal (22). Sirt1 is a nicotinamide adenine dinucleotide-dependent deacetylase that is associated with healthspan and longevity and inhibits the development of aging-associated diseases (23). Moreover, Sirt1 protects against aging-related neuronal degeneration and cognitive decline (24). Sirt1 affects a variety of biological functions, including DNA repair, energy metabolism, and mitochondrial homeostasis (25). There is increasing evidence that elevated Sirt1 activity can have beneficial effects on aging and aging-associated diseases. For example, Kim *et al*. revealed that resveratrol reduced neuronal degeneration in models of Alzheimer’s disease through activation of Sirt1 (26). P53 as a tumor suppressor plays an essential part in the aging and activation of P53 which induces apoptosis or cellular senescence (27). Moreover, if DNA repair is unsuccessful, P53 protein leads to apoptosis and cell cycle arrests (28). On the other hand, P53 is unstable and expressed at low levels under normal circumstances (29). Sirt1 via increasing deacetylation of P53, inactivated this protein and reduced the P53-dependent functions (30). P21 is one of the first downstream targets of P53, and it is an essential mediator for P53-dependent cell-cycle arrest (31). In this study, we found that P53 expression was significantly higher and Sirt1 expression was significantly lower in the aging model than in normal controls. Moreover, AT significantly decreased P53 and P21 expression and increased Sirt1 expression as well. The beneficial effects of AT on Sirt1, P53, and P21 mRNA expressions and levels were investigated. AT reduced the cognitive impairment induced by a high-fat diet and neurotoxicity induced by amyloid β peptide via Sirt1 activation (32, 33). Moreover, AT reduces the expression of P53 in spinal cord injury induced by chronic fluorosis (34). Accordingly, aging activation of the P53/P21 pathway can be attenuated by AT and it may be due to the Sirt1 deacetylation of P53.

Aging-associated pathological conditions contain many aspects including oxidative stress damage, destructed cells, and dysfunction of organs (35). Moreover, The roles of oxidative stress and ROS in aging have been well characterized (7). On the other hand, it has been demonstrated that the antioxidant activity could be beneficial for slowing this aging process (6). Studies have shown a significant decrease in antioxidant defense in DG-induced aging animals (11, 36). Consistent with these studies, we observed that aging decreases expression of CAT and GPx. Our results also showed that AT more potentially at the dose of 1 mg/kg restores the expression of CAT, SOD, and GPx mRNA compared with the aging group. Many studies have indicated that AT has a potent antioxidant activity. In our previous study, we found that AT at the dose of 1 mg/kg significantly increased the level of brain SOD compared with DG-treated mice (16). Mehrzadi *et al*. revealed that AT enhances the activity of renal antioxidants such as SOD and decreases renal ROS level in gentamicin-induced nephrotoxicity (37). Another study, demonstrated that AT could decrease ROS in endothelial cells via enhancing the enzymatic activity of thioredoxin (38). Moreover, Another study showed that AT reduces lipid peroxidases and increases SOD in 6-hydroxydopamine-induced dopaminergic toxicity in rats (39). Hence, the reduction of antioxidant enzymes may be responsible for DG-induced increased oxidative stress, and AT attenuates the increased oxidative stress parameters.

In this study, we showed that chronic treatment of DG increased the expression of Bax as well as decreasing the expression of Bcl-2 in the brain. As we discussed previously, DG administration increases ROS levels in the brain which leads to increased and decreased expression of Bax and Bcl-2, respectively (40). This subsequently releases cytochrome C to the cytoplasm and induced neuronal apoptosis via caspase-3 activation (41). The above-mentioned pathologic pathway is an important component of neurodegeneration in brain aging (35). We also found that administration of AT at 0.1 and 1 mg/kg doses reverses the mentioned pathologic events. It is well documented that AT has anti-apoptotic properties. He *et al*. revealed that AT protects against contrast-induced nephropathy via inhibiting the apoptotic pathways (42). In another study, AT showed its protective effects by reducing the Bax/Bcl2 ratio in a stroke model induced by middle cerebral artery occlusion (43). Accordingly, the beneficial effect of AT in aging may be associated with the anti-apoptotic pathway.

Besides oxidative stress, with age the production of inflammatory cytokines increased and seems to have a detrimental role in the process of aging (44). It is well documented that D-gal administration promotes the production of ROS that activates inflammatory pathways. Following long-term DG treatment, a variety of inflammatory cytokines such as iNOS, COX-2, IL-1β, and TNF-α are released in the aged animals’ brains (45). Moreover, it has been demonstrated that microglia activation via overproduction of ROS stimulates the release of these inflammatory mediators (46). TNF-α is one of the most important components of the inflammatory responses which induced the synthesis of inflammatory mediators (47). TNF-α induces the synthesis of nitric oxide and prostaglandins by expressing iNOS and COX-2 via NF-κB activation (48). Our present study showed that DG administration for 6 weeks up-regulated the mRNA expression of inflammatory iNOS, COX-2, IL-1β, and TNF-α in mice brain, while AT co-administration (more potentially at dose of 1 mg/kg) with DG attenuated mRNA up-regulation of these inflammatory biomarkers. Multiple lines of evidence demonstrated the anti-inflammatory effects of AT in different pathological conditions. AT attenuated the fatty liver-induced memory dysfunction via decreasing TNF-α as well as increasing BDNF levels in the hippocampus and prefrontal cortex (49). Furthermore, AT reduced inflammatory mediators (COX-2 and iNOS) as well as inflammatory cytokine levels (TNF-α and IL-1β) in the dorsal root ganglion and spinal cord injuries induced by chronic constriction (50). Thus, AT had an anti-aging effect and may be a potential candidate for treating aging-related diseases but future studies are needed to further clarify the exact mechanisms involved.

It is well established that during aging, the BDNF level reduces, which is associated with cognitive impairment (51, 52). Various studies demonstrated that the expression and level of BDNF decreased in both natural and accelerated models of aging (53, 54). We also found that chronic administration of DG decreased the mRNA expression of BDNF. Moreover, we demonstrated that AT at the dose of 1 mg/kg attenuated the distractive effects of DG on the mRNA expression of BDNF. These results are in agreement with our previous study which showed AT increased the BDNF level in aging mice induced by DG (16). On the other hand, previous reports revealed the beneficial effect of AT on BDNF expression. It was reported that AT enhances brain plasticity following a stroke in mice via increasing the mRNA expression of BDNF (55). In another study, it was shown that AT improves functional recovery in stroke patients by increasing the BDNF level (56). So, there might be a possibility that increased BDNF level contributes to the AT restorative effects.

## Conclusion

The results of the present study confirm our previous findings in the anti-aging effects of AT at the gene level and clarify further the mechanisms involved in this effect. 

## Authors’ Contributions

MM and IF conceived and designed the experiments. NTM and MHN performed the experiments. NTM, FK, and IF analyzed the data. IF and MHN contributed reagents/materials/analysis tools. MM, FK, and IF wrote the paper. All the authors read and revised the manuscript.

## Conflicts of Interest

The authors declare that there are no conflicts of interest.
